# What makes weekend allied health services effective and cost-effective (or not) in acute medical and surgical wards? Perceptions of medical, nursing, and allied health workers

**DOI:** 10.1186/s12913-017-2279-z

**Published:** 2017-05-12

**Authors:** Lisa O’Brien, Deb Mitchell, Elizabeth H. Skinner, Romi Haas, Marcelle Ghaly, Fiona McDermott, Kerry May, Terry Haines

**Affiliations:** 1 Department of Occupational Therapy, Faculty of Medicine, Nursing and Health Sciences, Monash University - Peninsula Campus, PO Box 527, Frankston, VIC 3199 Australia; 2 Allied Health Workforce, Innovation, Strategy, Education and Research Unit, Monash Health, Moorabbin, Australia; 3 Western Centre for Health Research and Education, Melbourne, Australia; 4 Allied Health Research Unit Kingston Centre, Monash Health, Melbourne, Australia; 5 Department of Social Work, Monash University, Melbourne, Australia; 6 Monash Health, Melbourne, Australia; 7 Allied Health Research Unit Kingston Centre, Melbourne, Australia

## Abstract

**Background:**

There is strong public support for acute hospital services to move to genuine 7-day models, including access to multidisciplinary team assessment. This study aimed to identify factors that might enable an effective and cost-effective weekend allied health services on acute hospital wards.

**Methods:**

This qualitative study included 22 focus groups within acute wards with a weekend allied health service and 11 telephone interviews with weekend service providers. Data were collected from 210 hospital team members, including 17 medical, 97 nursing, and 96 allied health professionals from two Australian tertiary public hospitals. All were recorded and imported into nVivo 10 for analysis. Thematic analysis methods were used to develop a coding framework from the data and to identify emerging themes.

**Results:**

Key themes identified were separated into issues perceived as being enablers or barriers to the effective or cost-effective delivery of weekend allied health services. Perceived enablers of effectiveness and cost-effectiveness included prioritizing interventions that prevent decline, the right person delivering the right service, improved access to the patient’s family, and ability to impact patient flow. Perceived barriers were employment of inexperienced weekend staff, insufficient investment to see tangible benefit, inefficiencies related to double-handling, unnecessary interventions and/or inappropriate referrals, and difficulty recruiting and retaining skilled staff.

**Conclusions:**

Suggestions for ensuring effective and cost effective weekend allied health care models include minimization of task duplication and targeting interventions so that the right patients receive the right interventions at the right time. Further research into the effectiveness and cost effectiveness of these services should factor in hidden costs, including those associated with managing the service.

## Background

Acute public hospitals are 24 h, seven days per week services, with continuous demand for care. Although hospitals aim to consistently provide high quality care, hospital staffing levels are lower on weekends than on weekdays, with resulting differences in the availability of diagnostic and/or treatment options [1]. A concerning temporal impact on the quality of patient care [2–4] called the “Weekend Effect” (whereby hospital patients admitted on weekends experience worse outcomes compared with those admitted on weekdays [5]) was recently confirmed by the Global Comparators Project [6].

Worldwide, there is strong public support for acute hospital services to move to genuine 7-day models, with senior clinical decision makers available at all times, and multidisciplinary team assessment of all newly admitted patients within 14 h of admission [7–9]. Proponents of 7 days a week allied health services argue that patients admitted on a Friday afternoon can face delays up to 72 h before assessment and treatment, leading to “extended periods of bed rest, decreased functional levels, reduced rehabilitation potential, limited discharge planning, and ultimately an increased length of stay” [10]. There is emerging evidence that earlier provision of specific allied health services improves health outcomes for some hospital patient populations [11–18]. There is also evidence that delayed access to some allied health services could result in higher rates adverse events [19–21].

There is a need to understand which aspects of weekend allied health services contribute to their effective (in terms of ability to achieve intended outcomes) and cost-effective (most efficient and least expensive) delivery so that efficient models of care can be employed. Two key stakeholder groups that may have insight into these factors are the staff providing these services and the multidisciplinary team (MDT) that works with weekend allied health services. They are uniquely placed to provide rich data on the benefits of the service that are not captured by standard hospital quality performance indicators. Their experience also enables them to identify inefficiencies or wastage of resources. To date hospital staff perspectives of weekend allied health services have not been explored, despite their first-hand experience. The aim of this study was to investigate the perceptions of MDT members regarding the effective and cost-effective delivery of these services on acute hospital wards.

## Methods

This qualitative study was conducted before commencing two stepped wedge cluster randomized controlled trials (RCT) involving 12 acute wards from two urban tertiary Australian public hospitals [22]. The first trial involved the sequential removal of the current weekend allied health service model from each participating ward in monthly intervals with services reallocated to wards not involved in the trial that previously had scant or absent weekend allied health service delivery. The second trial involved the roll-out of a new stakeholder-driven model of weekend allied health service delivery in the same random order. This meant that each trial ward had no weekend service for 7 months, and the reallocation wards had 6 months of gradually increasing - followed by 6 months of gradually diminishing - weekend service. The scope of weekend services provided at participating hospital wards before the study commenced is presented (Table 1).

**Table 1:** Existing hours of weekend cover per allied health profession for included wards at both included hospitals

	Hospital A		Hospital B	
	Saturday	Sunday	Saturday	Sunday
Physiotherapy	8	12	6–8	6–8
Occupational therapy	3	3	4	_
Speech pathology	3	3	_	_
Dietetics	3	3	_	_
Social work	0.5	0.5	_	_
Allied health assistant	4	4	_	_

Staff working on the trial and reallocation wards, including weekend allied health staff, were invited to participate, as their involvement had focused their perceptions regarding the relative value of the service, making them important informants. Focus groups were conducted over an 8-month period with medical, nursing and allied health professional staff from all trial wards in the month prior to withdrawal of weekend allied health service. If individuals were unable to attend the group (as was the case for allied health staff who only worked weekend shifts) individual telephone interviews were arranged. We also conducted focus groups with staff from all three re-allocation wards in the month prior to new services being added.

Investigators experienced in conducting qualitative and participatory action research (LO, FM, DM, and RH) facilitated these. The teams interviewed in this study were composed of a combination of medical, nursing, physiotherapy, occupational therapy, speech pathology, social work, dietetics, podiatry, and allied health assistants who worked both independently and in conjunction with one another [23]. All teams had a Nurse Unit Manager (NUM) who coordinated overall patient care, and a designated medical and allied health lead. Ethical approval for the study’s procedures was obtained from the human research and ethics committees at Monash Health and Melbourne Health, and after being supplied with written and verbal information about how data would be stored, analyzed, and presented, all participants provided informed written consent, which included permission to record the interview and disseminate de-identified findings. All research procedures complied with the Helsinki Declaration.

Twenty-two focus groups were conducted, ranging from 30 min to 1 hour. All focus groups (apart from at Hospital A where two groups were conducted as part of larger medicine meetings and were solely comprised of medical staff) took place in the participating ward and included a mix of nursing and allied health staff. Two ward-based MDT focus groups at Hospital B also included medical staff. As focus groups were scheduled on weekdays to maximize participant numbers, we also conducted semi-structured telephone interviews with 11 weekend allied health workers (four physiotherapists, three speech pathologists, an allied health assistant and one each from occupational therapy, social work, and dietetics).

### Procedure

The research team developed a semi-structured question guide based on a review of the existing literature and in consultation with the participating hospitals’ site liaisons for the RCT (DM, ES). Questions aimed to explore participants’ perceptions and experiences of weekend allied health service on their specific ward, with probes used as needed to elicit more detail (see Table 2). Where individuals worked across two or more wards, they were asked to comment only on their experiences on the ward in which the group was being conducted. All groups and interviews were digitally recorded, and uploaded to nVivo software version 10 (QSR international) for coding.

**Table 2:** Semi structured interview questions

1.	What are the current duties/activities performed by the weekend allied health service? (Map the service on the ward). Why are they performing these roles?
2.	What do you think are the advantages of having a weekend allied health service? What sorts of patients on your ward most benefit from your current weekend allied health service and how? What makes it effective or cost effective?
3.	What are the disadvantages of the weekend allied health service? What do you think are the main threats to effectiveness or cost effectiveness?
4.	Are there particular allied health services that are most valuable at the weekend for your ward, eg OT, Physio, SW?
5.	Are there any duties/activities performed by weekend allied health staff that could be could be done earlier in the week?
6.	Are there any duties currently performed by weekend allied health staff that could be done by others who are present during the weekend (e.g. nursing, medical staff)?
7.	Are there any duties/activities performed by weekend allied health staff that could be put off until Monday?
8.	What concerns do you have about the withdrawal of the weekend allied health service? What is the reasoning behind these concerns? Are they based on evidence, experience or something else?
9.	Do you have any suggestions about how the weekend allied health service could be improved?
10.	How easy or difficult do you think it would be to change procedures in order to make these improvements?
11.	Are there any other comments you would like to make?

All groups and interviews had a facilitator and, where possible, a note-taker and an observer. After each group, the facilitator, note-taker and observer met to discuss overall impressions, emerging themes, and areas for further exploration. Question lists were subsequently amended as required, so that new themes could be explored in more depth.

### Data analysis

To develop an in-depth understanding of factors that participants thought impacted positively or negatively on the effectiveness and cost-effectiveness of weekend allied health services, we used thematic data analysis principles to identify patterns of meaning across the data set. This commenced with the previously mentioned meetings immediately following the focus groups to ensure that important constructs or ideas were not missed. Data were collected and analyzed in an iterative manner allowing for emerging themes to be further explored in subsequent focus groups or interviews. Three authors (LOB, DM, and RH) independently conducted microanalyses of the group recordings and field notes in nVivo with LOB and RH developing a codebook based on initial reading and discussion of the data. Thematic analysis’s approach to coding the data was followed, using first level coding (describing and categorizing responses), second level coding (identification groups in the first level codes - e.g. these are all types of barriers to cost-effectiveness), then constructing themes that reflected and described a coherent and meaningful pattern in the data [24]. We met several times to analyze coding categories, review potential themes, then define and name emergent themes until saturation [25]. When researchers identified a snippet of audio that clearly encapsulated a specific code or theme, it was transcribed verbatim. Member checking of themes was conducted during presentations at forums in both hospitals in order to add to the trustworthiness of findings. There were no instances of participants disagreeing with the themes during these forums, however some were further expanded or explained in more depth.

### Background, training, and preconceptions of investigators

All investigators were from an allied health background and were primary investigators for the RCT evaluating the effectiveness, cost-effectiveness and safety of the existing weekend allied health service as well as a new stakeholder driven model [22]. None were currently working weekends, although four had done this previously. Prior to the study commencement, the investigators anticipated concern from MDT members about the quality of patient care resulting from the temporary withdrawal of weekend allied health services from their ward.

## Results

### Sample characteristics

A total of 210 MDT members participated, including 17 medical staff (9 consultants), 10 NUMs 15 associate NUMs, 72 nurses, 29 physiotherapists, 19 speech pathologists, 17 occupational therapists, 17 dietitians, 11 social workers, two allied health assistants, and one podiatrist.

**Fig. 1:**
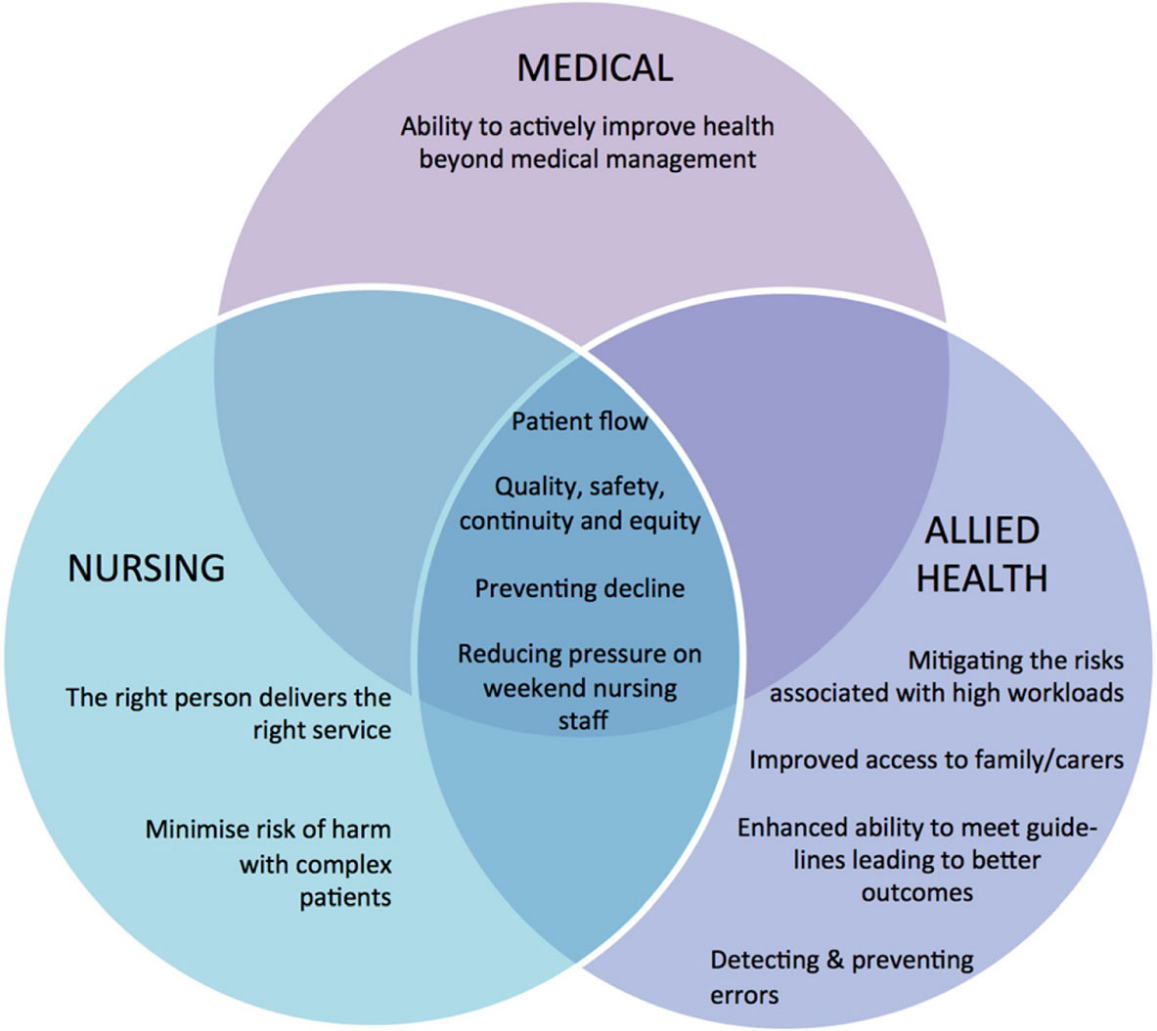
Issues considered to enable a more effective or cost-effective weekend allied health service by professional group

**Fig. 2:**
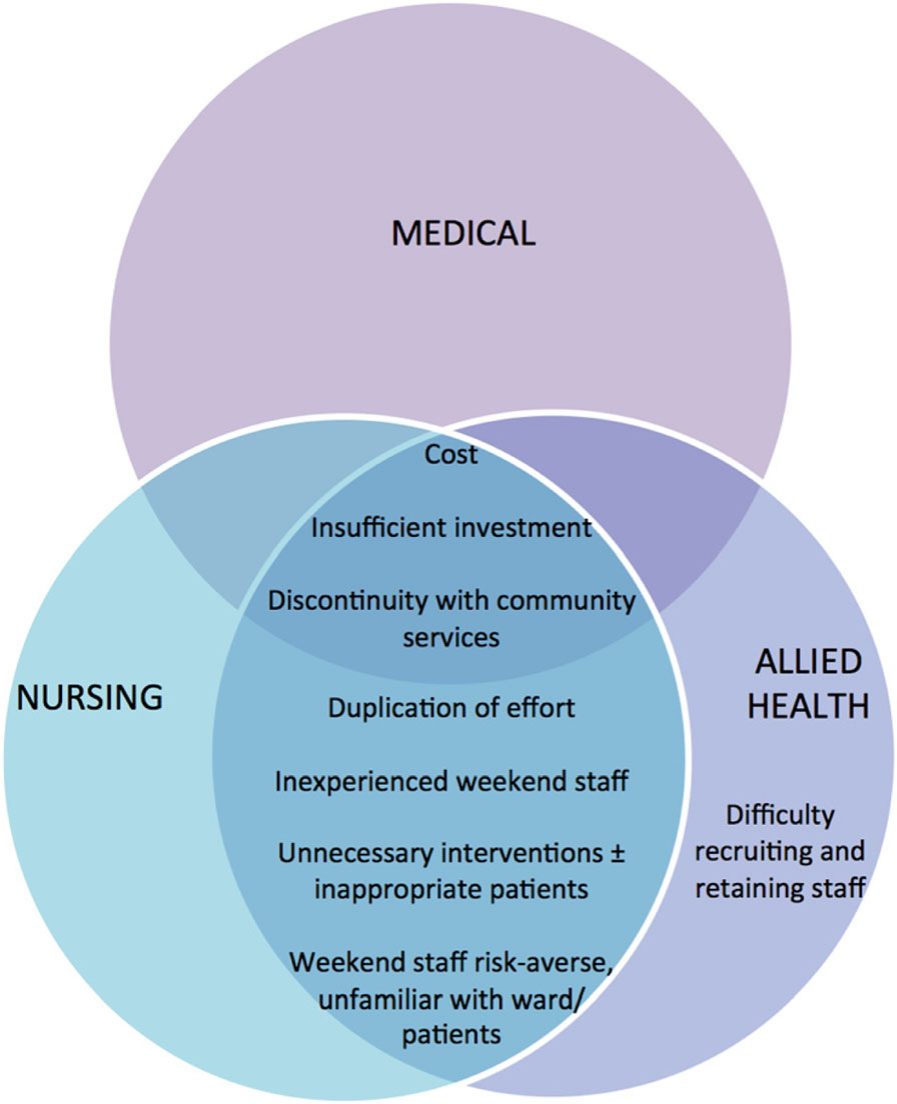
Issues considered as barriers to effective or cost-effective weekend allied health service by professional group

The key themes identified during the analysis are presented diagrammatically in Figs. 1 and 2. These have been separated into issues perceived as enablers of a more effective or cost-effective service overall (Fig. 1) and those that were barriers (Fig. 2). Both of these figures further separate these issues according to the professional groups that raised and discussed each issue.

## Themes common to all staff regarding the enablers of effective and cost-effective delivery of weekend allied health services

### Patient flow, effects on length of stay, and resulting management pressure

All participant groups expressed the belief that the presence of weekend allied health services positively contributed to patient flow through the hospital, and that length of stay would increase without it. Some group participants raised the point that there was little evidence available that has examined whether these services do actually reduce length of stay. However, this point did not appear to change the conviction held by many group members that these services are effective for reducing length of stay.

*Nurse (Hospital A): I know for a fact that we need allied health, and I know it (the removal of services as part of the trial) will affect discharges and patient safety.*

*Occupational Therapist (Hospital B): the aim is to shorten the length of stay, so there are no gaps in patients' care…Earlier intervention allows earlier effectiveness of treatment and again facilitates discharge.*

Reducing length of stay was viewed as being important for several reasons. First to reduce the cost of service delivery per patient, but also to enhance the ability of other patients to access acute care services, and to prevent poorer health outcomes associated with longer than necessary length of stay.

*Nurse (Hospital B): it would be a shame to have a patient here that didn't really need to be, and sick people in the Emergency Department and ambulances backed up… I'm hoping (this trial) doesn't have that sort of effect.*

*Occupational Therapist (Hospital B): Nobody wants to be in hospital longer than they need to be, in terms of that emotional well being and feeling like they're progressing. We don't want to keep them here any longer than they have to be.*

When probed further about the consequences of exceeding the average length of stay, some staff commented on budget pressure or punitive action from more senior managers. Having a weekend allied health service was seen as being protective in this regard.

*NUM (Hospital A): the repercussions are I have to keep going back to these meetings (with executive management) and feeding back to these guys....*

*Speech Pathologist (interrupts): We have targets for length of stay and if a patient stays longer than their target …*

*NUM (interrupts): we lose money. After a certain amount of days we lose money, yes.*

### Quality, Safety, and continuity: Equity of patient care

A shared sense of concern regarding quality of care was threaded through all focus groups and interviews. This was sometimes expressed as concern that patients would be exposed to higher risk of adverse events in the absence of weekend allied health.

*Speech Pathologist (Hospital A): when it comes to understanding and making decisions or life-changing recommendations, (they need to be) based on an assessment that comes from a very informed background. Just like it wouldn't be very appropriate for us to start doing the doctors' work!*

*Nurse 1(Hospital B): If a patient has had a decline on Friday evening … if we don't have a speech pathologist clearing them as safe to eat or drink, they'll stay nil orally until Monday.*

*Interviewer: what are the consequences of being nil by mouth for that long?*

*Nurse 2: malnutrition*

*Nurse 3: For some of the (nil by mouth patients) the doctors might be reluctant to put a (nasogastric tube) down because of advanced age or dementia*

*Nurse 1: and on a weekend that decision won't be made. The after-hours covering doctor will say "we'll wait" and put intravenous fluids off until the speech pathologist (assesses them) on Monday.*

The concern about quality of care was also articulated as an equity issue, in that patients admitted on a weekend or late on a Friday would receive poorer standards of care than those admitted early or mid-week.

*Nurse (Hospital A): I suppose I find it stressful because to us, other than there is less staff around, the weekend is exactly the same. We still do theatre Saturday (and) Sunday; our patients are still sick all weekend. We still need just as much expert help on the weekend as we do during the week. Nothing actually changes. We don't close down beds; we don't stop operating. Saturday and Sunday is exactly the same as Thursday and Friday.*

### Preventing functional decline

The majority of nurses felt they rarely had time to perform more than core duties on the weekend, and it was perceived that complex patients were more likely to be left in bed instead of mobilizing if they had not undergone physiotherapy assessment.

*Physiotherapist (Hospital A): (without weekend allied health) the patient just sits there - it's like two days rest, and it just puts them back again.*

*Medical staff (Hospital B): They’re sitting in bed all day. You think about someone who’s quite old and hasn’t got much muscle anyway, they’re likely to decondition more easily especially over the weekend.*

### Reducing pressure on weekend nursing staff

All staff felt that weekend nurses were under significant workload pressure, considering the lower levels of weekend medical and other staff.

*Medical staff (Hospital B): There's a skeleton medical staff (on the weekend) and that can consume some extra nursing time, making it quite difficult for people to take on extra things*

*Nurse (Hospital A): I think we have enough to do. I think we already have too much to do! I see nursing in a rush. I see cut corners everywhere. I see quality of patient care replaced with quantity, and I see this increasing.*

*Nurse (Hospital A): The physio getting (the patient) out of bed for the first time is important to us, because that is very time consuming for us (nurses) to do.... it gives us that time back to put back into our own clinical work.*

Whilst most nurses agreed that workloads were manageable, some felt that quality could be compromised leading to poorer patient health outcomes.

*Nurse (hospital A): most nurses pick up jobs, and try to do the best they can within the time and effort they've got, but it doesn't always work to the betterment of the patient. We're just not well enough qualified in that area; we do the best we can but we don't always make the best decisions*

## Themes specific to Medical staff

### Ability to actively improve health of patients during their admission

Medical staff argued that the benefits of medical care alone were limited if not combined with activity, the primary domain of allied health. Whilst this may not result in immediate discharge, the results might be seen later in the patient’s admission.

*Medical staff (Hospital B): if you get the patients up from day one, their lung function improves; they get better. It's not just preventing; we admit patients to hospital, and if we sit them down for three days in a hospital bed doing nothing, we're not actually doing anything for three days other than parking them in hospital with an oxygen tube and having some intravenous (medication) or whatever.*

*Medical staff (Hospital B): all of allied health is very handy over the weekend, but the one we feel the most is definitely Physio. … We find a lot of our patients who are at high risk tend to go a bit backwards over the weekend quite a lot of the time, or at least don’t go forward. That holds up discharge and makes everyone’s life a bit more difficult.*

## Themes specific to nursing staff

### The right person delivers the right service

It was clear that nurses valued allied health expertise in assessment and intervention, and this was highlighted on weekends when there was less access to experienced medical staff.

*Nurse (Hospital B): if you have junior (medical staff), or an intern on, and the registrar is really busy, and you have a patient who's a bit chesty it is a huge help to have a physio there to listen to their chest and help facilitate the care for that patient. That's more important on the weekend because we don't have all the medical staff on that we normally would.*

Respect for specific allied health skills was also expressed in terms of professional boundaries with some nurses very clear about which tasks lay outside their own scope of practice.

*Nurse (Hospital A): We don't have the necessary skills to know which is the appropriate gait aid to use so we'll just mobilize patients with two nurses rather than with the appropriate gait aid. (But the risks of this are) patients falling and nurses injuring themselves.*

In contrast, some nursing staff felt able to take on some tasks seen as the traditional domain of allied health.

*Nurse (Hospital B): Doctors want physios to do chest physio and I say to them, “We do chest physio, saline nebulizers, we do active breathing cycles, what further do you want? That patient is coughing and able to expectorate, they are doing bubble PEP, do we need to have physio on top of that? We can do that.”*

*Nurse (Hospital A): if a nurse sees that the patient is having trouble in the shower or getting out of bed, we don’t have to wait for the physio or the OT, we can do that. We can make a decision. We can say, “yes, he walks safely” or “he can manage at home”.*

Extending from this respect for professional expertise, nurses’ comments revealed an underlying sense of security that came from being able to draw on allied health input over the weekend.

*Nurse (Hospital A): I think as a general nurse on the weekend, just to have the feeling of security, I guess. If you're having a physio or somebody available for whatever needs you've got, you know that you're having the proper assessment done, so that the patient's not at risk of falls or whatever. You know, you're going to have a proper gait aid … chosen for the patient and all the forms are documented and done properly. It gives you the security that you've done the right thing to prevent an injury.*

This was also evident in some nurses expressing a lack of confidence in reporting domestic violence or abuse issues, preferring to rely on the expertise of the social workers.

*Nurse 1 (Hospital A): (Social Workers) seem to know the right way to refer or the right avenue to refer our patients to the appropriate (services after discharge). Some things they can do that we as nurses cannot do.*

*Nurse 2: Like domestic violence issues, child protection issues. They are very, very important. We get patients here who have been bashed or abused or something like that, and the patient's not safe to go back home. That's where the social worker is able to find that out and do something about it.*

### Minimize risk of harm with complex patients

It was apparent that, while most nurses were confident about undertaking some allied health tasks, this was only the case when the patient was seen as “uncomplicated” and the risk of an adverse event, such as a fall, aspiration/choking, or staff injury, was perceived to be low. Challenging previous recommendations made by medical or allied health, even when patient circumstances had clearly changed, was perceived by some to be especially risky.

*Nurse 1 (Hospital B): It's just the discharges, from a legal side of things. If on a Friday the physio says "not safe for discharge" and then the next day they're walking around on their own, you know, where do you sit with that if they're doing everything for themselves but someone has written down "not safe for discharge"?*

*Interviewer: How confident would you feel about reviewing that decision, considering the change in the last 24 h?*

*Nurse 2: I think that's a seniority thing. I'd be happy, but …*

*Nurse 1: sometimes the doctors say, " When cleared by allied health". We're not allied health.*

*Nurse (Hospital B): What if, for example, (the patient) needs a shower stool and he's a young fit guy who's been on crutches for 6 weeks? Oh sure I can hand over a shower stool, but if he fell in that bathroom and came back in with a head injury, that concerns me. It's really not my qualification; you do years of university to be an occupational therapist and it is their specialty. Even though, in the back of my head I'm thinking, “this is all really simple”, there's something there saying, "I'm taking this responsibility,… this ownership; I'm then educating the patient, and I've now taken responsibility for this"… It's beyond my scope of practice.*

## Themes specific to allied health

### Mitigating the risks associated with high workloads on Mondays and Fridays

In all focus groups, allied health staff commented that Mondays and Fridays were the busiest days of the week, with the highest risk of patients being missed or receiving very abbreviated services. There was a shared perception that the presence of the weekend service mitigated this risk.

*Occupational Therapist (Hospital B): Fridays are a bit the same because you're not (going to be) here for 2 days, so there's this sense in the department that Fridays and Mondays are the worst days of the week.*

*Physiotherapist: if you pick up a new patient at 3.30 on Friday, and there are issues, that's when you think "Thank God there's a weekend service so I can get them to follow them up".*

### Improved access to family/carers

Allied health staff with weekend experience noted that liaison with family members was often easier on weekends, and that this enabled them to provide a more comprehensive handover to carers.

*Social Worker (Hospital A): A lot of time the families work from Monday to Friday… within the hours that we work. If we have (weekend staff)… within that time we can get (the family) to come in, (so we can) teach them about (things like) transfers, and equipment.*

*Occupational Therapist (Hospital A):(on a weekend) we can talk to the patient and their carers and relieve any anxiety, provide information about what's available, talk about what they can link in with during the week and how they can do that.*

### Enhanced ability to meet care guidelines leading to better health outcomes

In some wards, allied health professionals were particularly keen to ensure that patient care adhered to hospital protocols or national standards. The weekend service was seen as essential to achieving this. The removal of services during the trial was concerning for some staff as protocols or standards might be breached which would expose both patients and the organization to the risk of poor health outcomes.

*Speech Pathologist (Hospital A): Just recently a patient had had a stroke … and 2 days later I found out! The doctors said that they could eat and drink and they had been, but I thought OK, just because they haven't coughed, it doesn't mean that they're necessarily tolerating their diet or fluid. So … that's a risk there. You want to be able to see the patient and make sure they're going to be safe*

### Detecting or preventing errors that would otherwise be missed

Allied health participants described performing tasks that they felt should be performed by medical staff but were not being done consistently. They felt that these tasks may be missed on weekends without an allied health service, leading to poorer patient outcomes.

*Speech Pathologist (Hospital A): The national stroke guidelines, which we adhere to, (recommend that) the full allied health team sees the patient within 48 h of admission, and the MRI or CT should happen within the first four hours. Often we're the ones identifying things and pushing (for) the CT. The doctors should be on to it, but it is often allied health advocating (for it).*

## Themes common to all staff regarding the barriers to effective and cost-effective delivery of weekend allied health services

### Financial cost

The primary barrier to cost-effectiveness identified by all groups was the relative costliness of weekend services compared to weekday services. In Australia, workers are paid penalty rates on weekends (up to twice the hourly wage) and may also receive extra annual leave entitlements after working a threshold number of weekend shifts. Participants recognized that having a weekend allied health service in addition to the traditional 24 h services of medicine and nursing, came at a cost to the health service which may not be justified by the improvement in health outcomes attained. They also recognized potential cost savings that could offset this high wage rate if these services were able to reduce length of stay.

*Physiotherapist (Hospital B): The cost of the service needs to be balanced against the savings they are making by discharging patients.*

### Insufficient investment to generate benefits

All groups expressed frustration regarding the inability of weekend staff to service all referrals on busy weekends, or to service individuals sufficiently to generate measurable benefit.

*Weekend Speech Pathologist (Hospital B): You don’t have enough hours and so you are just doing the clinical risk stuff and that won’t have an impact on the length of stay because you’re not getting to the functional maintenance stuff.*

Some medical staff questioned the value of having low levels of weekend allied health cover, as this could mean that assessments were cursory and did not result in real progress in the patient’s condition or movement toward discharge.

*Medical staff (Hospital A): (Weekend allied health) are possibly a little more time-pressured, and unable to guarantee to be able to do as comprehensive a review of the patient’s issues.*

*Medical staff (Hospital B): If it’s not funded well, there is a service but it’s not doing what it could potentially do –it’s only got a very limited coverage*

### Discontinuity with community services on weekends

All groups were aware that the reduced availability of community services such as meals on wheels, home help, and homeless shelters restricted the ability of allied health staff to facilitate discharge.

*Medical staff (Hospital B): You just can’t push out certain cohorts of patients because other institutions, other facilities, other services don’t have (weekend) provision. So it works well for some patient cohorts, ones who perhaps, can go home independent or with family support. You can’t do that for every ward and every patient, so it’s about picking the right ward and the right patient …. Some you can’t move them on. It doesn’t matter if you see them on a Saturday or Sunday and deem them safe, they are still not going to go.*

*Social Worker (Hospital A): one of the difficulties with discharge at the weekend might be that we can’t contact service providers on the weekend so if you have a service that needs to start on the day that the person is discharged, they may not be appropriate for a weekend discharge. Or in terms of social work and liaising with community, that might be something that can’t be done on the weekend.*

## Themes common to nursing and allied health

### Duplication of effort

Ward staff reported that at times this meant that patients were sometimes ‘double handled’ in that they were seen by the weekend allied health team who could not complete the discharge plan, and some work had to be repeated on the Monday by the weekday team.

*Nurse (Hospital A): A lady who was about to be discharged asked to see a social worker, so we called the social worker on call but they couldn’t arrange anything because there is nothing open on the weekend anyway. So then that still has to wait until Monday before it can be sorted out.*

*Dietitian (Hospital A): I am (often) referred patients (at risk of refeeding syndrome) just to check bloods and I think, “that’s not a good use of our service”. If pathology are picking up that someone’s potassium is super low, they don’t need a dietitian as the middleman – they can go straight to the doctor.*

*Dietician (Hospital A): If someone starts something on the weekend, then we have to reassess anyway in terms of interpretation and things like that*

### Employment of under-skilled/inexperienced staff

Participants identified that weekend roles tended to be filled by less experienced staff to cover weekend shifts. They felt that these staff might not have the equivalent skills, work rate and decision making confidence to expedite patient discharges. This was not a criticism of weekend allied health services in general, rather the particular approach to implementing it at that particular hospital.

*Physiotherapist (Hospital B): staff that work on the weekend are junior… and they don’t have the experience to decipher (the urgency of a referral) with a quick read of the notes – they’ll take an hour and write a page of notes where that could have been done on a Monday.*

### Unnecessary interventions, inappropriate referrals, or both?

Some weekend allied health workers openly questioned whether what they did actually helped with outcomes such as length of stay or prevention of adverse events.

*Physiotherapist (Hospital B): Does (our intervention) actually have an impact or not? How do we know that we are providing the best care by seeing them day one post-op or not? It's really making me question whether the physio referrals, or the patients that we were seeing, whether they were appropriate or not. Or whether we need to carefully look at our referral criteria … and what patients should be seen on the weekend.*

They questioned the appropriateness of interventions they were providing (“was it necessary at that time, was it necessary at all?”) and the appropriateness of the patients they were being referred to see.

*Physiotherapist (Hospital B): I think there is a lack of clarity about what kind of patients should be referred to the weekend physio service and we often get completely inappropriate referrals and then don’t get the people that should be (referred).*

Allied health staff all operated to department-based “priority tools” which direct the order in which patients should be seen. For example, patients due for discharge that day were highest priority, as were those at risk of serious deterioration likely to result in an admission to Intensive Care. These priority tools were not always known to nursing staff.

*Nurse (Hospital B): If (the physio) is busy elsewhere, then … I'm feeling that they're saying they won't come because they're low on the priority list. So what IS the priority list from physio's point of view?*

*Interviewer: and that's not clear to you - how they priorities patients?*

*Nurse: No. Clearly if (a patient) came out of ICU, then yes they would need to see them if they have a tracheostomy. … Otherwise … sitting them out of bed, ambulation, that (seems to be) low on their priority.*

### Weekend staff who are risk-averse, unfamiliar with the ward or patients

Another theme common to nurses and allied health was that weekend staff often came from a separate staffing pool to the weekday staff. This was perceived as less efficient, in that staff needed time to familiarize themselves with the patients listed for them, and they did not have the awareness of contextual issues or available resources that could only be gained by attending weekday ward or team meetings. In some cases, even when familiar with the patient, they were still unwilling to make decisions they perceived as risky.

*Occupational Therapist (Hospital A): We have staff that work here on the weekends that don’t work during the week. We need to be very careful with clinical handovers to get the job finished off. Weekend staff are often from different organizations so don’t know our procedures all that well*

*Nurse 1 (Hospital A): They don’t really know the patient so they need our input – lots of our input - to make a decision and sometimes they can’t make a decision. They leave it for the next day*

*Interviewer: so they hold it over to the weekday staff?*

*Nurse: I’ve seen it happen – they can’t make decision even if the (weekday) physio has said, “OK for discharge” they say, “I don’t know! I think we’ll wait for tomorrow”…they don’t work here during the week; …they don’t want to take a risk.*

## Themes specific to allied health

### Difficulty finding and keeping weekend staff with the right skill mix

Allied health staff frequently commented on the need for a pool of experienced staff with broad-ranging and current skills to cover weekend shifts. This was seen as challenging to achieve, especially when one staff member covers the entire hospital.

*Speech Pathologist (Hospital A): Our clinicians on the weekend have to understand the whole gamut of patients because they are working hospital wide – it’s hard to get adequately skilled staff across the continuum.*

Retaining staff, ensuring adequate supervision, and maintaining currency of skills was also seen as a challenge. Provision of supervision and training for weekend staff was felt to be extremely difficult and reviewing documentation was often regarded as the only way to audit performance. Supervision structures for weekend staff were perceived to be weak compared to that for weekday staff.

*Speech Pathologist (Hospital A): Weekend staff do not participate in mandatory training and so feel quite fragmented and removed from the rest of the team. From a safety and a “roundness’ of experience, having access to training would be fantastic…. Some may be stay at home mums during the week and the last training they went to was 5 years ago.*

In Hospital B, where training was provided, having to frequently replace and train workers was a problem that contributed to the overall cost of the service.

## Discussion

The issues perceived to contribute most to the effectiveness of weekend allied health services were the belief in positive impacts on patient flow, functional independence, and quality of care. Whilst there is emerging evidence in support of this in sub-acute care, there is limited evidence in acute care [22]. A recent systematic review found weak evidence in support of weekend physiotherapy services following knee arthroplasty in the acute setting [26], but other studies in acute populations have had unclear results possibly due to issues with study design [27, 28]. For sub-acute populations, one randomized controlled trial found that weekend physiotherapy services were likely to reduce overall length of stay in cognitively intact, rehabilitation patients [29]. Another meta-analysis [30] of pooled individual data from two randomised controlled trials involving stroke patients [31, 32] identified a significant improvement in length of stay for those receiving additional weekend therapy when analyses were adjusted for hospital site, age, walking speed and Functional Independence Measure score on admission. The applicability of these results to acute settings may be limited due to the substantial difference in length of stay.

Interestingly, most participants were aware that there was limited evidence supporting weekend allied health services in acute care, but this did not influence their belief in its effectiveness. This could present a barrier to the translation of research findings should the results of future trials show that the service is not effective. This finding is consistent with other research that has found that many clinicians are still placing greater emphasis on their own clinical experience and the influence of colleagues than they are on relevant research findings [33–36].

A theme we identified was “the right person delivering the right service.” This theme highlighted a perception amongst nurses that there were some tasks that were not adequately skilled to perform. These findings are consistent with other qualitative studies of nurse perceptions [37–39]. It can be argued that this is reasonable, given the evidence relating to allied health’s effect on preventing some adverse events. For example, there is level 1 evidence from a meta-analysis of randomized controlled trials that non-invasive ventilation, an intervention commonly provided by physiotherapists [21, 40, 41], reduces in-hospital mortality and prevents intubation in specific “at risk” populations [20]. Perceived practice boundaries can, however, be challenged for other tasks that may arise on weekends such as mobilizing patients whose recovery is uncomplicated, reporting domestic violence or abuse issues, providing basic aids or equipment for discharge, or arranging post-discharge services. These were all tasks that allied health participants thought nurses had the capability to do (and, in the case of abuse, are mandated to do), but about which many nurses expressed a lack of confidence or competence. It is possible that improved competency and credentialing structures including training could improve nurses’ capacity to undertake these tasks, although it is unclear how these tasks would be prioritized among existing nursing duties.

The issue of role boundaries appears linked to lowered risk tolerance amongst nurses. Some nurses expressed fears of being held responsible for adverse events if they made decisions that were outside their actual or perceived scope of practice. This could be symptomatic of a deeper issue termed “moral distress”. This occurs when professionals cannot provide what they think is best for the patient [42] and is considered a major problem in the nursing profession [43]. It is reported to be common when there are insufficient staffing numbers and/or inadequately trained staff, both of which can occur over weekends. Socially structured defense mechanisms in response to moral distress can include eliminating decisions by ritual task performance, reducing the weight of decision-making responsibility by checks and counterchecks, redistribution of responsibility to other professionals, and reducing the impact of responsibility by delegating decisions to superiors [44]. This was evident in the present study when nursing staff deferred decision making to allied health.

Allied health professionals may also experience moral distress in this context, as ensuring that patient care meets minimum guidelines and standards for the purpose of accreditation (e.g. Australian Commission on Safety and Quality in Healthcare [45]) emerged as a key theme, consistent with other research [46]. Where allied health staff feel that weekend services are inadequate to meet these standards, for example management of the deteriorating patient, this could influence retention, sick leave and the mental health of staff members [47, 48]. This was the case even in the absence of evidence of efficacy and cost-effectiveness.

Financial costs, staffing issues, and inefficiencies relating to double handling, or lack of impact of assessments delivered by inexperienced staff were identified as factors that may render these services inefficient. Previous research conducted in rehabilitation wards found that Saturday allied health services saved AUD$41,825 per Quality Adjusted Life Year gained [49], however this evaluation did not take into account costs associated with managing the service, or other costs such as time taken for weekday staff to provide handover information to weekend staff. Suggestions to improve effectiveness and cost-effectiveness in acute medical and surgical wards include using skilled staff from other areas that do have a weekend allied health service (e.g. Emergency or Intensive Care Units) or implementing an on-call staffing model rather than employing weekend allied health staff in fixed shifts.

### Strengths and weaknesses

This study recruited large numbers of acute medical, nursing and allied health staff across a variety of disciplines in two tertiary health services, although the data may not be generalizable outside of Australian settings. Some of the issues related to weekend services in general, however others related specifically to the models employed at the research locations. It is possible that involvement in the RCT influenced the perceptions of participants, as they were aware that the service was being removed for seven months, and then would be reinstated in a different form. This is likely to have been perceived by participants as a threat to ongoing funding, resulting in a defensive response and an exaggeration of benefits of the service and potential risks of withdrawing it. Their opinions may therefore differ from those held by staff at other hospitals whose service was not at risk of being withdrawn. This may, however, have led to deeper reflections on the strengths and weaknesses of the existing service as participants were also informed that their input would help shape the new stakeholder driven model of weekend allied health services.

## Conclusions

Weekend allied health care models would benefit from identification and minimization of duplication of tasks. They also need to ensure that the right patients receive the evidence-based interventions at the right time in order to be effective and cost effective. More evidence is needed to determine the effectiveness and cost effectiveness of these services, however it is important to note that since each hospital arranges services differently, results may not apply across varying settings. Future evaluations of weekend services need to appropriately factor in some of the hidden costs that we have identified in this study. These include costs associated with managing the service, recruiting, training and replacing staff, and time spent by weekday staff writing referrals for weekend lists that could otherwise be spent treating patients.

## Abbreviations

LOS: Length of Stay
MDT: Multidisciplinary team
NUM: Nurse Unit Manager
RCT: Randomized Controlled Trial
